# Supplementary figures and images for: Axonal T3 uptake and transport can trigger thyroid hormone signaling in the brain
Source: eLife. 2023 May 19;12:e82683. doi: 10.7554/eLife.82683 (PMC10241515; doi:10.7554/eLife.82683)

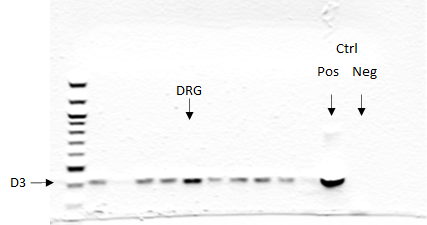

Supplement: Figure 1—figure supplement 3—source data 1. [file elife-82683-fig1-figsupp3-data1.zip › Blots/FigS2-D3-blot-labeled.tif]

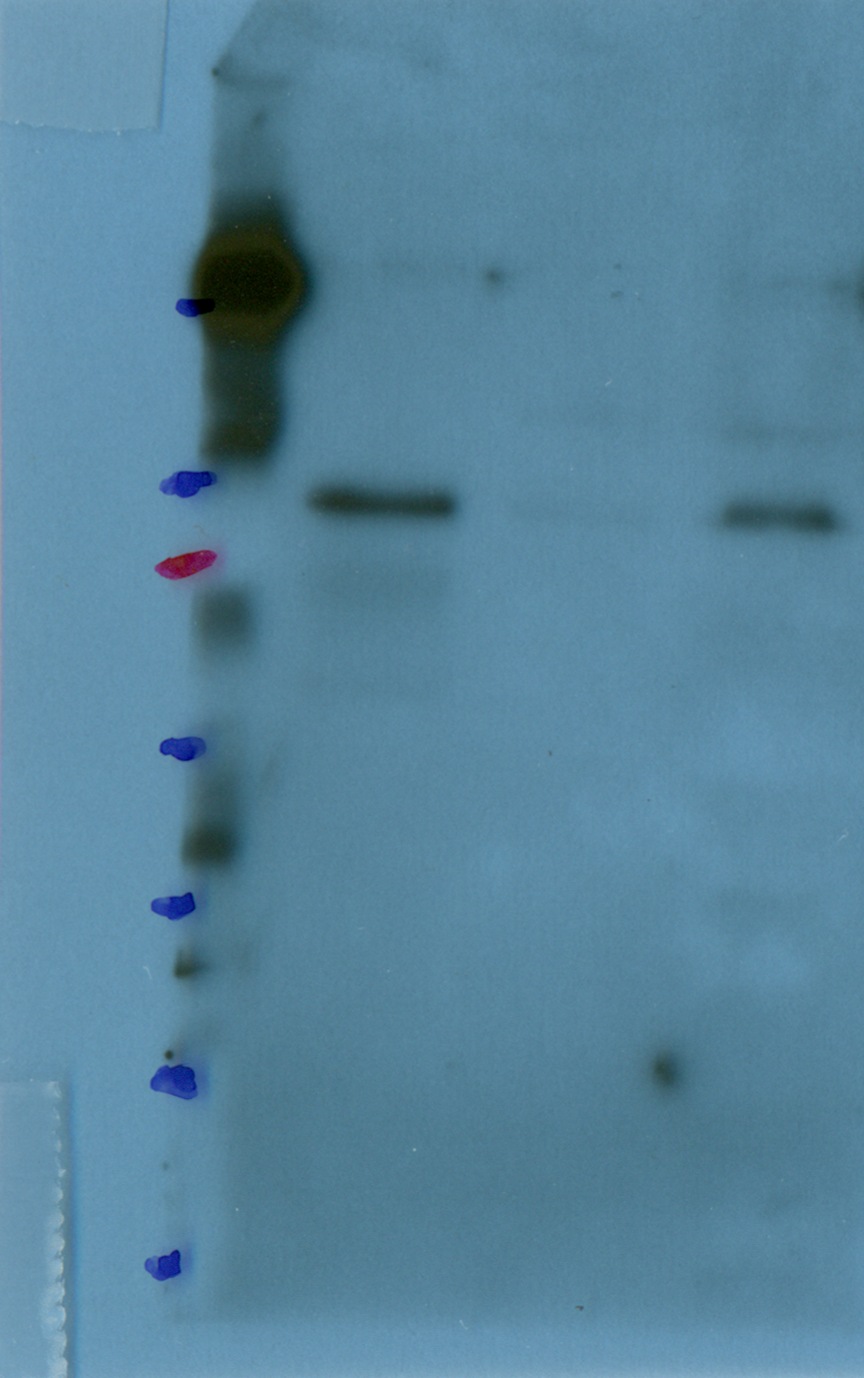

Supplement: Figure 1—figure supplement 3—source data 1. [file elife-82683-fig1-figsupp3-data1.zip › Blots/FigS2-MCT8-blot.tif]

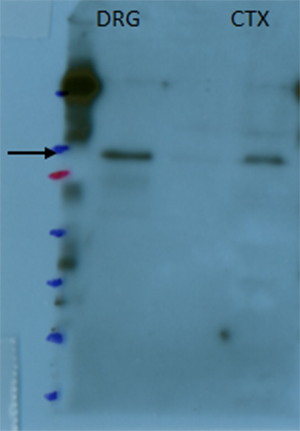

Supplement: Figure 1—figure supplement 3—source data 1. [file elife-82683-fig1-figsupp3-data1.zip › Blots/FigS2-MCT8-blot-labeled.tif]
